# Understanding how neglected tropical diseases programs in five Asia-Pacific countries adjusted to the COVID-19 pandemic: A qualitative study

**DOI:** 10.1371/journal.pntd.0012221

**Published:** 2024-05-30

**Authors:** Alison Jaworski, Adam T. Craig, Clare E. F. Dyer, Julio Goncalves, Nalisa Neuendorf, Jamee Newland, Angela Kelly-Hanku, William Pomat, David MacLaren, Susana Vaz Nery

**Affiliations:** 1 The Kirby Institute, UNSW Sydney, New South Wales, Australia; 2 School of Population Health, Faculty of Medicine and Health, UNSW Sydney, New South Wales, Australia; 3 Centre for Clinical Research, Faculty of Medicine, The University of Queensland, Queensland, Australia; 4 World Health Organization, Dili, Timor-Leste; 5 Papua New Guinea Institute of Medical Research, Goroka, Papua New Guinea; 6 College of Medicine and Dentistry, James Cook University, Cairns, Queensland, Australia; Shandong Provincial Institute of Dermatology and Venereology, CHINA

## Abstract

**Background:**

Following the COVID-19 pandemic declaration, the World Health Organization recommended suspending neglected tropical diseases (NTD) control activities as part of sweeping strategies to minimise COVID-19 transmission. Understanding how NTD programs were impacted and resumed operations will inform contingency planning for future emergencies. This is the first study that documents how South-East Asian and Pacific NTD programs addressed challenges experienced during the COVID-19 pandemic.

**Methodology/Principal findings:**

Data was collected through semi-structured interviews with 11 NTD Program Coordinators and related personnel from Fiji, Papua New Guinea, The Philippines, Timor-Leste, and Vanuatu. Constructivist grounded theory methods were drawn on to generate an explanation of factors that enabled or hindered NTD program operations during the COVID-19 pandemic.

The COVID-19 pandemic disrupted NTD programs in all countries. Some programs implemented novel strategies by partnering with services deemed essential or used new communications technology to continue (albeit scaled-back) NTD activities. Strong relationships to initiate cross-program integration, sufficient resources to implement adapted activities, and dedicated administrative systems were key enabling factors for recommencement. As the COVID-19 pandemic continued, exacerbating health resources scarcity, programs faced funding shortages and participants needed to find efficiencies through greater integration and activity prioritisation within their NTD units. Emphasising community-led approaches to restore trust and engagement was critical after widespread social anxiety and disconnection.

**Conclusions/Significance:**

Sustaining effective NTD programs during a global emergency goes beyond managing immediate activity disruptions and requires attention to how NTD programs can be better ensconced within wider health programs, administrative, and social systems. This study underscores the importance of pre-emergency planning that reinforces NTD control programs as a critical service at all health systems levels, accompanied by governance arrangements that increase NTD staff control over their operations and strategies to maintain strong community relationships. Ensuring NTD units are supported via appropriate funding, personnel, and bureaucratic resources is also required.

## Author summary

Disruptions to health services during the COVID-19 pandemic may delay progress in neglected tropical diseases (NTDs) control by several years. Current studies of how NTD programs responded to the challenges arising from the COVID-19 pandemic are largely from African settings and focus on earlier periods during the pandemic when policy advice was most stringent. In contrast, this study documents how NTD staff and other related personnel in five South-East Asian and Pacific countries adapted to changing situations throughout the pandemic. We found a difference in the strategies NTD programs initially used to manage the immediate challenges of strict new public health measures compared to those used later in the pandemic’s evolution when seeking to recommence usual programs. Initially, some NTD programs devised new ways of offering modified services, primarily though cross-program integration with other health services that were allowed to continue operating. When operations re-commenced, NTD programs faced financial challenges that meant human and resource efficiencies in these units needed to be found. The cumulative impact of pandemic-related restrictions and misinformation eroded community trust in health programs more broadly and required NTD staff to rebuild local relationships. The lessons learned in these settings may help NTD programs in these and other regions prepare for prolonged future emergencies.

## Introduction

Neglected tropical diseases (NTDs) are a group of 20 diseases of poverty impacting 1.74 billion people world-wide, yet have historically received relatively little funding, political attention, or research and technical support [1–3]. Despite recently increasing efforts to control these diseases—including mass drug administration (MDA) campaigns, enhanced surveillance, water, sanitation, and hygiene (WASH) programs, and morbidity management—NTDs remain significant public health problems in endemic settings [3–5].

Countries in the South-East Asia and Pacific regions are affected by a diverse range of NTDs, including vector-borne diseases such as lymphatic filariasis (LF), illnesses caused by parasitic helminths such as schistosomiasis and soil-transmitted helminths (STH), and by bacterial infections such as trachoma [1,6]. Some countries have made significant progress in controlling NTDs as a public health problem; for example LF and trachoma in Vanuatu [4,7] and LF, rabies, and leprosy in The Philippines [3]. The management of other endemic NTDs—such as scabies in Fiji and Vanuatu; schistosomiasis and food-borne trematodes in The Philippines, and STH across the regions–pose ongoing challenges [6,8].

With the World Health Organization (WHO)’s declaration of COVID-19 as a pandemic on March 11, 2020, many South-East Asian and Pacific countries pursued a suppression strategy, involving extended international and domestic border closures, adoption of ‘testing-tracing-and-isolation’ procedures, alongside restrictions on social movement and gatherings, including school closures [9,10]. In April 2020 the WHO recommended that core NTD activities—including population surveys, MDA, and active case finding—be suspended to reduce the risk of COVID-19 transmission associated with these large-scale community-based events [11].

Mathematical modelling predicts that the disruption to NTD programs resulting from suspensions or reductions in activities during the pandemic may have set progress on NTD elimination back by up to 2–5 years [12,13]. Further, the World Bank estimates an additional 6–8 million people in the region were pushed into extreme poverty as a result of the economic impacts from the COVID-19 pandemic [14]. As well as increasing populations’ vulnerability to NTDs, slow global economic recovery is expected to exacerbate the pressures on domestic health systems, potentially undermining earlier gains in universal health coverage and infectious disease control [9,15–17].

In July 2020, (three months after recommending suspension of NTD programs) the WHO issued precautionary measures to guide NTD activity resumption under a risk-benefit assessment framework given local capacity to deliver effective and safe interventions within the pandemic context. Measures recommended included implementation of social distancing and enhanced infection prevention and control (IPC) practices while implementing NTD activities [11]. There is some indication that such measures have been useful in facilitating short-term recommencement of activities such as MDA [18–22]. However, these reports come from a limited number African countries at a time when many NTD programs were leveraged for pandemic control efforts, including case finding and the contribution of resources to hand hygiene and COVID-19 community awareness-raising initiatives [17,20,23]. Such experiences may not be generalisable, particularly in the Pacific region which was largely free from community-transmission during the first year of the pandemic [24]. Whilst the use of strong suppression strategies was effective in minimising COVID-19 mortality particularly in western Pacific countries compared to other regions generally [9], the impact of these strategies on local NTD programs apart from the overall disruptions reported globally is not clear [25]. Furthermore, later developments in the COVID-19 pandemic, such as the vaccination rollout and the eventual relaxation of many public health measures as part of a general shift towards ‘living with the virus’ [9], are likely to have generated new challenges for NTD programs.

This research aims to provide new perspectives on how NTD programs in the South-East Asia and Pacific regions responded to the COVID-19 crisis as well as to generate insights that will lead to better preparation for future pandemics and other major disrupting emergencies.

## Methods

### Ethics statement

This study has been approved by the University of New South Wales Human Research Ethics Advisory Panel D: Biomedical (HC210183) and relevant national Ministry of Health ethics review boards: the Fiji Human Health Research and Ethics Review Committee (FNHRERC 19/2021); Timor-Leste Institute National of Health-Research Ethics & Technical Committee (INS-RETC: 1500); Vanuatu Ministry of Health Ethics Committee (no number); the Papua New Guinea Institute of Medical Research Institutional Review Board (IRB #2015) and the Papua New Guinea Medical Research Advisory Committee (MRAC 20.35). National ethics approval was not required for the Philippines.

### Study design

This study was informed by constructivist grounded theory (CGT), a qualitative analysis approach that aims to generate an explanation empirically derived from real-world events [26,27]. CGT is widely used to study interactions between individuals and their social contexts, when analysing new problems, or where there are few existing theories or well-developed explanations about a phenomenon [28]. The COVID-19 pandemic provides a prime example of this kind of situation.

### Participant recruitment and selection

Five countries in the South-East Asia and Pacific regions with endemic NTDs and where NTD control activities are in place were selected. These were Fiji, Papua New Guinea, The Philippines, Timor-Leste, and Vanuatu. NTD unit personnel–or those with responsibility for NTD activities if there was no NTD unit in their country–were purposely selected to participate in the study. Individuals were eligible if they were (i) responsible for implementing, managing, or supporting NTD programs during the COVID-19 pandemic and (ii) able to discuss COVID-19-associated impacts on NTD program delivery. Invitations to participate in the study were circulated through the *Australian Centre for the Control and Elimination of NTDs (ACE-NTDs)*, a network of public health researchers and other personnel throughout Australia, Asia, and the Pacific, to seek recommendations and assistance in contacting potential participants. If a suitable person was identified and agreed to participate, individuals were contacted by the research team and asked to confirm their participation by returning a copy of the full participant declaration statement. For two respondents who did consent to be interviewed but where the complete statement was not supplied, informed verbal consent was documented by the interviewer via audio recording according to the approved study protocol before interview commencement.

### Data collection instrument and procedures

A semi-structured interview format was chosen as this allows flexibility to explore issues raised by interviewees whilst also covering desired topics [29]. An interview guide (S1 File) was developed that included questions related to five key areas. These were (i) key NTDs of concern and control activities typically undertaken; (ii) impacts of COVID-19 on NTD control activities; (iii) program responses to challenges faced in the delivery of NTD services during the pandemic; (iv) modifications to WASH programs as part of the pandemic response and the associated impact of these on NTD programs; and (v) lessons learnt from the COVID-19 experience that will inform future program design. However, due to the separation of WASH and NTD units within the countries interviewed, participants were largely unable to provide specific details of impacts of the COVID-19 pandemic on WASH programs that would inform the research objectives.

Data collection commenced in June 2021 but was paused in October 2021 as the continuing deployment of potential participants to the COVID-19 pandemic response left little opportunity for engagement in non-essential activities. After a 9-month suspension, data collection resumed from June to September 2022. Prior to recommencement, the interview guide was updated to support data collection related to the evolving situation (S1 File). Also, during this time we attempted to contact participants who had been interviewed in 2021 to seek further insights in relation to emerging findings.

Most interviews were conducted using videoconferencing technology (Zoom or MS Teams) and in English by AJ, CD, and NN, all of whom have prior experience in health-related qualitative or NTD research. In-person interviews with participants from Timor-Leste were performed by JG who has local health research experience and who translated the interview schedule from English to Tetum (the local language). The duration of interviews ranged from 50 minutes to two hours. All interviews were audio-recorded, transcribed, and translated when required by a member of the research team or independent transcription service.

### Data analysis

Data coding was completed by the first author and involved the generation of inductive categories through repeated readings and recoding of data using the constant comparison method [27]. This involved: (i) the generation of codes inductively from the data through the examination of processes, concerns, meanings and assumptions conveyed by the participants; (ii) grouping these codes under concepts or key ideas according to shared properties; (iii) the further refining and consolidation of key concepts through the constant comparison of different pieces of the data, participants and timepoints for similarities and differences in order to generate broader theoretical categories or themes about the experience [30,31]. Similar to the process outlined by Cheer and colleagues [32], analysis of earlier interviews commenced whilst data collection was still being undertaken. Emerging findings (such as changes in key challenges over time, increasing impacts of strained community relationships) were then followed up in subsequent interviews [31]. Regular meetings were held between authors (AJ, AC, DM, SVN) to review this process and critique the emerging analysis developed to explain participants’ experiences.

Data analysis was undertaken using NVivo v12 (QSR International Pty Ltd). De-identified quotes extracted from interview transcripts for this paper have undergone minor editing to enhance readability.

To facilitate full and frank information sharing, participants were asked whether they gave permission for verbatim quotes to be included in published findings, with or without attribution to the individual’s role and country. Seven of eleven participants gave their permission for quotes to be used with attribution. The presentation of results here reflects the differing permissions granted, although to ensure participants’ anonymity, we have used non-specific role descriptions and/or country descriptions in place of the attribution details consented to in some instances.

## Results

We conducted 12 interviews with 11 NTD program coordinators and related personnel from five countries: Fiji, Papua New Guinea, The Philippines, Timor-Leste, and Vanuatu. Five persons were interviewed in 2021: two persons from Fiji, two persons from The Philippines, and one person from Vanuatu. In 2022 six interviews took place, with one person from Papua New Guinea, three persons from Timor-Leste, and two additional participants from Vanuatu. Another participant who had been interviewed in September 2021 also agreed to be re-interviewed in August 2022. Four interviewees were Program Coordinators, and seven were NTD Managers. For the purposes of this study, the term ‘Program Coordinator’ refers to officers usually with responsibility for a particular disease (e.g., Lymphatic Filariasis Program Coordinator) whilst the term ‘NTD Manager’ refers to persons in senior roles that oversee wider NTD teams/personnel including NTD unit managers, WHO NTD focal staff and National Disease Control Directors. As most countries in the study have very small NTD programs, further role detail is omitted in order to protect participant identity. Seven participants had between two to five years’ experience in their current role, two participants had between five to ten years’ experience, and two participants had over 10 years’ experience.

**Fig 1:**
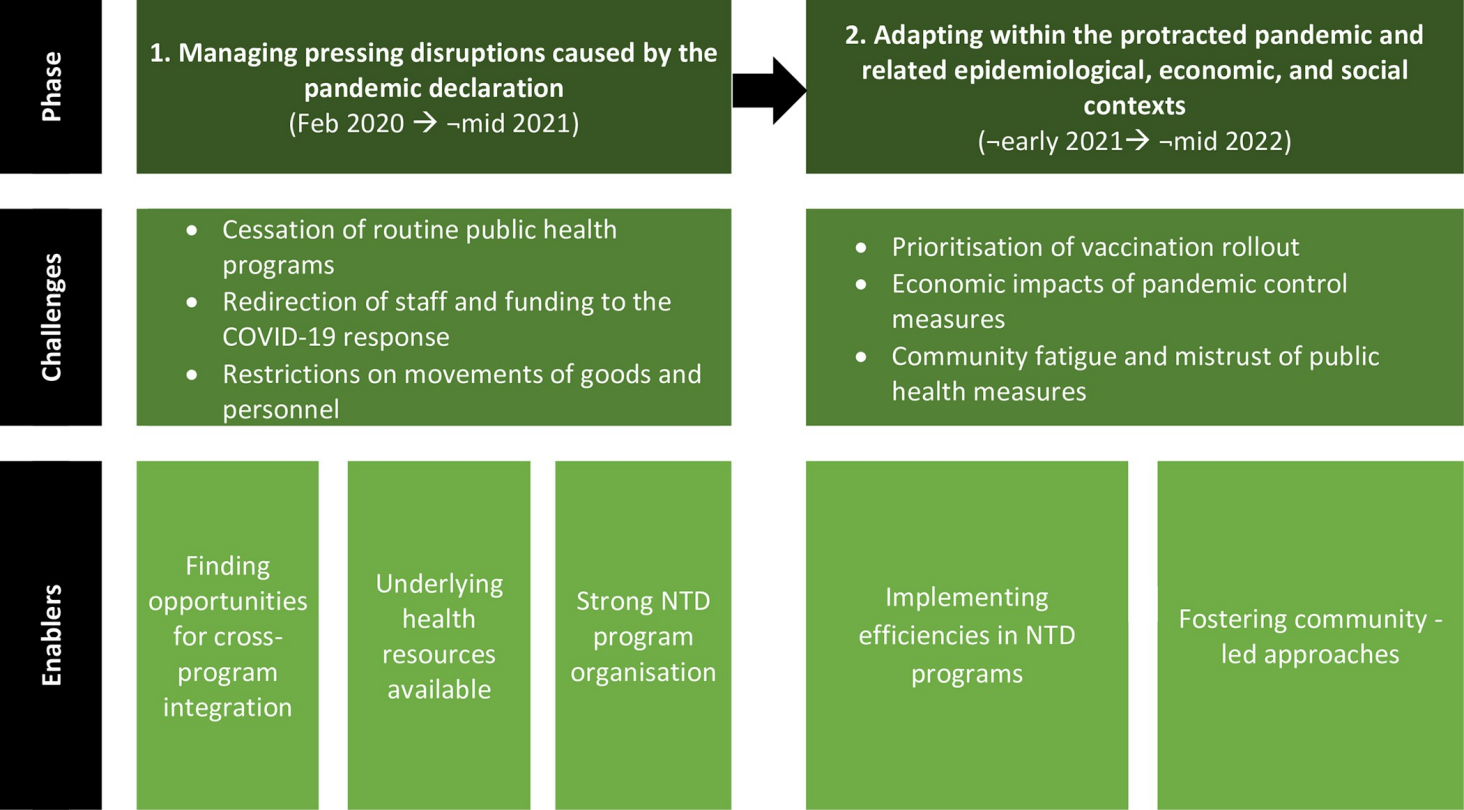
Model of how NTD programs were impacted by the COVID-19 pandemic and elements that enabled resumption of control and management activities.

Participants described NTD programs as moving between two distinct phases during the pandemic, influenced by the timing of key global and local events including the introduction of emergency public health measures, international border closures, the rollout of COVID-19 vaccines, as well as the cumulative effects of these developments (Fig 1). The following results are presented by phase.

### Phase one: Managing pressing disruptions caused by the pandemic declaration

Contextual information about country specific NTDs of concern provided by participants is summarised in S1 Table. Participants agreed that their NTD control activities were affected either upon or shortly after COVID-19 was declared a pandemic. This occurred across all settings regardless of the existence or level of local COVID-19 transmission. Subsequently, and for approximately 12–18 months, some activities–typically MDA–were reported as suspended completely, although most interviewees also noted that surveillance, case finding, treatment, and morbidity management for NTDs in general was significantly curtailed. Participants explained that movement restrictions imposed to minimise COVID-19 transmission meant that it was not possible to deploy centrally located staff to implement NTD control activities, while also disrupting medication and supply chains and limiting access to health workers and services across both regions. Many participants gave specific examples as to how personnel and goods transportation restrictions halted planned programs:

“*We managed to implement [LF control plan] in three municipalities but when COVID-19 came…because of the sanitary fence*, *we could not implement it*.*”* (Participant 10)

“*…and then even during that time the domestic flight was closed*, *so we have to wait…and then ship some medicine down*” (Participant 2)

Health systems across countries were reported as having been underprepared for a health emergency of the scale of the COVID-19 pandemic. Respondents from various countries commented that improved preparation would have enhanced service delivery continuity during the unprecedented restrictions and when widespread diversion of financial, human, and other resources to COVID-19 outbreak responses was occurring.

“*If I was given again the chance to go back*, *I would be more resilient and proactive in coming up with some sort of activities or strategies that would really address ‘if anything happens like this’*.*”* (Participant 3)

“*There’s so many good lessons that we’ve taken out of the emergency response in terms of what we needed to make available*, *whether it’s manpower*, *on the ground*, *SOPs …so that they can make sure that*, *you know*, *there are still services that are continuing*.*”* (Participant 8)

Program Coordinators and NTD Managers in multiple settings identified that in the face of these pressures, the relatively low power position held by NTD programs in the health bureaucracy hierarchy meant they had little control over decisions being made even while these impacted usual NTD service delivery and care. This was evident in participants’ expressions of their powerlessness when responding to questions about alternative avenues to continue programs.

“*…unfortunately we cannot make it [deliver programs] because the decision has been made from the [senior management] level to cease all the [NTD] activities*.*”* (Participant 7)

“*… all the funds that [were] supposed to be used for NTD services*, *are… diverted to [the] COVID response…[so] how can you implement because there’s no more funds*.*”* (Participant 3)

While interviewees appreciated the rationale for program cessation or wind-back in the early stages of the pandemic, there were concerns about the long-term impacts on public health arising from disarrangement to established NTD control plans. This sentiment was expressed more strongly among participants from Pacific countries–which were comparatively less affected by COVID-19, at least in the initial stages of the pandemic–than those in South-East Asia. As one Pacific interviewee explained:

“*We’re trying to reach the elimination target*, *here…[But] at the end of the day*, *I understand also…because you don’t have the human resources*. *And because we are a less mortality rate disease…the priority goes more towards COVID-19…”* (Participant 5)

### Finding opportunities for cross-program integration

In multiple settings, cross-program integration–the integration of NTD activities within the delivery of other health programs–was reported to allow NTD staff to continue to reach affected populations whilst working within COVID-19 public health movement restrictions. By sharing and leveraging off other health programs’ human and service delivery resources, NTD Managers described how they were able to maintain community awareness campaigns, respond to local outbreaks, and conduct surveillance and case follow-up–albeit in at a drastically reduced scale.

“*Another innovation is we work through MOH’s Family Health Program… [that] already exists in every municipality…we contact them to do follow up*, *observe the patient’s progress and report to us*.*”* (Participant 9)

Participants noted that establishing cross program integration models was opportunistic and depended on if other programs had resources and managerial/ bureaucratic support to continue operations. The ability to integrate NTD activities into other programs was reportedly enabled by strong personal and professional relationships, levels of goodwill across programs, and broad staff awareness of the importance of NTD program continuity for its success. One participant explained this when recounting how a joint endeavour between an NTD program and a childhood measles immunisation campaign in a Pacific Island country facilitated the continuance of scabies treatment to children under five years:

“*…through the integration approach that we have…we’re still able to reach the community that has a high number of scabies… And also the understanding that we have between the programs to work together…Because if the other program’s not willing to work together that is also a challenge*.*”* (Participant 2)

Furthermore, several participants commented that without established connections and mutual goodwill, there was little that could be done to facilitate cross-program integration opportunities. NTD Managers noted that they rarely had the ability to direct personnel or resources from other programs that were necessary to meaningfully integrate NTD control activities within the other parties’ scope–particularly in health systems that had decentralised responsibility for these decisions at district levels. One participant explained this in terms of efforts to supply deworming drugs within the schoolwork packages distributed to households following school closures in a South-East Asian country, when describing the problems this approach faced due to lack of local capacity:

“*What we really observed when we shifted MDA services to the community was that it didn’t make a difference… So*, *despite our support and assurance that you have to still continue these NTD services*, *it really depends on them–if they have the time*, *the ability*, *and the resources*.*”* (Participant 3)

### Underlying health resources available

The capacity to implement interventions modified according to COVID-19 IPC guidelines was noted as a pre-requisite to resuming NTD program delivery by multiple participants. Financial, human, and technical resources were required to implement these IPC measures, but these were not uniformly available in some settings. For example in Timor-Leste, one interviewee described being able to continue NTD monitoring visits subject to their own negative COVID-19 test. However another participant had explained that the country had initially needed international support to develop the laboratory facilities capable of managing such widespread COVID-19 testing:

“*Before we didn’t have [laboratory capacity] to test and detect a virus like this [COVID-19]*. *But now we have capacity to test more than 2000 samples per day and … we also have a genome sequencer that can do analysis in-country*.*”* (Participant 9)

Some participants were able to utilise telecommunications technology to maintain team communication and monitoring activities, to continue oversight of patients requiring morbidity management, or to deliver staff training remotely. However, these examples were rare and were typically discussed in the context of centrally located health departments or major health facilities where adequate technological resources were in place. One participant from a Pacific Island country who had given an example where information and communications technologies had been used to support clinical management during MDA prior to the pandemic, stressed that successful delivery of NTD programs required attention to the surrounding context in which these programs were situated. They felt that the pandemic had highlighted the importance of a whole of health systems approach including a well trained workforce, reliable supply chain, and supportive management systems overseeing frontline services:

“*You need the right trained people*, *you need the drugs…available at the facilities*, *you also need to make sure that testing*, *diagnostics are available*. *So it’s not just…one part of the system*, *it’s the whole work of the system that will make it possible to achieve elimination… And if you have things in the middle that are not working*, *oiling the [machine]…it’s stuck*, *you need to make sure that the wheel is actually turning*…*”* (Participant 8)

### Strong NTD program organisation

Having well organised NTD program operations in place prior to the pandemic was one important factor that allowed staff better access to the necessary bureaucratic, funding, and operational decision-makers that enabled activity resumption. In particular, interviewees from countries with a designated NTD unit appeared to be able to leverage their recognised position within the Ministry of Health (or equivalent) hierarchy to advocate for NTD programs’ continuity and resourcing. One participant from Vanuatu described how working together as an identified NTD team was helpful after a long period of funding delay:

“*…[we] email them*, *push them to talk … [we have meetings where] we discuss with them*, *that we need them to process our finance because we need to carry [out] our activities… and when they understand all that*, *they release the funds*.*”* (Participant 6)

In contrast, NTD programs were described as less visible and receiving of attention from decision-makers when they had been subsumed under wider infectious disease programs. One participant recounted how this structure made it more difficult for NTD staff to find avenues to advocate for and justify refocusing of attention on the resumption of NTD programs.

“*I think the plan there was to form the NTD [unit] and try and get it under just one program on its own it*, *so that it can get a lot more*, *visibility*, *etc*. *But while they were working on that… when COVID came in there was still focus in the ministry on [three priority communicable diseases]*, *but not NTDs*. *It wasn’t included”* (Participant 4, Interview 1)

### Phase two: Adapting within the protracted pandemic and related epidemiological, economic, and social contexts

As the pandemic progressed, some of the initial problems faced, such as inability to travel and NTD drug supply interruptions, were resolved. However, participants across all countries identified that funding environments, service delivery, and stakeholder/community relationship landscapes remained altered or new problems emerged.

Participants reported that sustainable financing for NTD programs was increasingly challenging, particularly as the economic disruptions caused by the pandemic put pressure on health budgets. Difficulties in capturing domestic and international attention for NTD programs continued, with participants now noting program and funder decision-makers increasingly prioritised the COVID-19 vaccine rollout as a necessary precursor to the lifting of public health restrictions. Consequently, some NTD Managers and Program Coordinators described how prolonged program disruptions made it difficult to demonstrate their impact and value for money, which then impacted their ability to secure ongoing funding:

“*In terms of funding availability*, *because the Ministry has its fiscal year from July to August each year…when pandemic hit us*, *there were some sort of delays in implementation*. *So*, *the reporting was late and the funding…it gets reverted back to the Ministry of [XXX]*, *or goes back to [the donor]”* (Participant 5)

Importantly, participants saw increasing community fatigue and frustration with COVID-19-related restrictions such as lockdowns led in some instances to disengagement and non-cooperation with health interventions more broadly. Participants noted this environment, along with misinformation spread through social media–particularly about the COVID-19 vaccination–fostered mistrust of health workers and undermined NTD workers’ relationships with some local communities. The consequences of these attitudes were experienced by participants in both South-East Asia and Pacific regions, several of whom reported being the target of verbal and physical abuse whilst in the community implementing routine programs or were informed about this occurring to colleagues:

“*They insult*, *swear at us*, *chase us away*, *but it happens on a small scale*. *It happens because they do not understand*…” (Participant 10)

“*They refused to take the medication [during MDA]*, *and even when the health staff or health team*, *goes to the community*, *they’re like*, *they’re attacking the health staff*.*”* (Participant 6)

In contrast, a participant in one Pacific Island country described high levels of engagement with COVID-19 interventions and vaccination programs, that afterwards translated into increased interest in and community capacity to support NTD programs. This participant attributed this success to health workers utilising explanations of disease management that aligned well with local socio-cultural understandings alongside clear upper management support for extensive community outreach:

“*The acceptance of health programs coming across to deliver service onsite is very high at the moment … and it’s something that our Ministry here has encouraged all health facilities to do*. *To leave the workplace and go to provide services to the community or to the doorstep*…*”* (Participant 4, Interview 2)

### Implementing efficiencies in NTD programs

Finding process and financial efficiencies in an environment of reduced funding was commonly mentioned in later interviews. Participants reported a need to be pragmatic and flexible regarding how they re-commenced NTD programs, given financial, personnel, and other constraints. In one Pacific Island country, a participant described how their team addressed these challenges by prioritising certain activities when resuming program delivery:

“*One of the first activities that we implemented was two weeks ago*. *So*, *probably*, *we’ll have …more activities during the upcoming MDAs…[But] we haven’t decided on anything apart from MDA activities yet*, *because we only have limited funding*. *Or otherwise…we would integrate MDA with NTD surveys*.*”* (Participant 7)

This quotation also highlights a shift in some participants’ language as the pandemic progressed from describing integration with other health programs (cross-program integration) to integrating NTD activities themselves (within-NTD program integration). Within-program integration was often discussed in the context of program sustainability. For example, one participant interviewed in late 2021 expressed that integration with other NTD activities should be prioritised in terms of how this could enable continued program viability by making use of increasingly limited available resources.

“*We’ll need more integration with other NTD program*. *I think that’s the only way forward…Funding-wise it’s hard…because our budget…it’s decreasing because of economic impacts*, *so… there we will need integration … For us to work on our own*, *that’s not going to be happening”* (Participant 5)

Indicated by these comments was the acknowledgement that NTD Managers and Program Coordinators would need to devise their own responses to the wider economic and political situation that affected funding flows. In Timor-Leste where support from an important external funder had just ceased, one participant explained how challenging this was without sustainable program financing. When discussing how integration could be used to help NTD programs become more efficient in tighter funding environments, this interviewee however suggested international donors should play a greater role, leveraging their financial power to assist NTD programs secure a greater share of domestic funding:

“*To solve those problems*, *all programs should be integrated because some programs have no budget*. *Now*, *each program has their own policy*, *so that the government can put money on that or other partners that want to support [the program] …[but] for example in the future a partner says that in this program ‘A’*, *we invest 50% and the other 50% by the government to implement the program*.*”* (Participant 11)

### Fostering community-led approaches

Participants also saw the pandemic as emphasising how community ownership underpinned the uptake and ultimate success of NTD interventions. For some, this required the strengthening of ‘bottom-up’ community-level approaches such as the provision of community-based education to address misunderstandings about NTD programs in the context of COVID-19, including vaccination concerns. Enhancing partnerships with trusted, local health workers or community leaders, who were able to shape information provision to suit the communities in which they lived, was seen to be increasingly important in fostering acceptance at this time:

“*We found out that instead of our side doing the awareness part*, *we can train [the local authorities] …then they go in the community*, *and because it’s their community*, *they know the community very well…so that they can understand the messages*…*”* (Participant 6)

However, as this and other participants noted, NTD programs needed to avoid simplistic education/awareness-raising that merely required village health workers and leaders to adapt externally created public health information. This was seen as particularly important if long NTD worker absences or the influence of misinformation had eroded community relationships, diminishing community gatekeepers’ support for any NTD interventions. Some participants instead felt that health worker-community relationships should prioritise giving communities a greater degree of input into how activities would be implemented, thereby increasing local ownership. For example, when discussing how program integration could support more effective resource use, one participant from a Pacific Island country noted that integration would be better placed if it was based on what combination of responses communities believed would most effectively address their needs across a range of diseases:

“*The way that our health system is designed is that we have our programs right at the top end …these programs are vertical and that’s where all these managers are integrating … It’s better that that integration comes from the community-based level here [gestures low]*, *they will know how to integrate it into the community*, *not from up here [gestures high]*.*”* (Participant 4, Interview 2)

Listening to local feedback from community was identified as a practice that created an enabling environment where contextually appropriate adaptations could be implemented. In one example of local adaptations, staff could re-interpret COVID-19 safe operating procedures in a low-risk setting to respond to community concerns and increase engagement:

“*This decision has been made from us … [due to] some of the feedback from especially the community health workers*. *Because they know that*, *if we wear PPE or masks*, *automatically these people will remember COVID and they will stay away from us*.*”* (Participant 7)

## Discussion

This study explored the impacts of COVID-19 pandemic restrictions on NTD programs operating in South-East Asian and Pacific countries, and how participants responded to maintain–albeit in reduced forms–surveillance, control, treatment, and morbidity management activities. These conversations provide insight into how challenges altered as the pandemic evolved, including how respondents needed to respond not only to service delivery constraints within pandemic containment measures, but also underlying health systems, financial, and social pressures exacerbated by a prolonged global health emergency. The implications of these findings for NTD programs, the regional health systems in which they operate, and the role international supporters can play when preparing for future emergencies are discussed below.

Throughout the study period, NTD Managers and Program Coordinators reported having little power to shape how their governments determined which health programs needed to be maintained, leading participants to find creative ways to continue program delivery. This power dynamic likely stems from the relative diminished value placed on NTD programs in complex epidemiological, economic, and social settings outside of emergency times [3,33]. As such, strengthening the bureaucratic, political, and social position of NTD programs as much as possible will need to be an important feature of any future emergency planning. Here we offer some strategies for consideration.

Flexibility to implement integrated COVID-19 public health responses has been helpful in fostering NTD control activity resumption in other settings, [17,18,22]. Our participants experiences’ offer examples of new ways of achieving resumption through integration with other programs deemed essential health services to leverage off their continued access to staff, community, and operating resources. However, these cross-program examples were a more ‘normative’ form of integration [34] that relied on strong working relationships with non-NTD staff who understood the need for NTD control initiatives and where senior management were open to distinct vertical programs working together. For such integration to be more ‘systemic’ [34,35], NTD program staff and decision-makers should proactively identify which other health programs would be supportive of cross-program approaches in advance, providing opportunities to develop (and test) governance and coordination arrangements, as well as the policies and standard operating procedures that would guide integration. As the dynamics of future emergencies are unknown, a ’principles-based’ design approach may be most appropriate and provide NTD programs a strong position from which to initiate future inter-program ventures should the need arise.

Whilst existing studies have focused on program-level modifications to facilitate the resumption of NTD programs during the COVID-19 public health emergency, [18,19,22] our research highlights the simultaneous importance of structural-level features. NTD programs were more promptly able to resume in settings where these diseases had a more prominent position within Ministry of Health structures–namely via a dedicated NTD unit. This emphasises the importance of recognising NTD programs as core activities of health authorities and ensuring they are supported by dedicated funding, staffing, and administration systems. While we acknowledge that it may not be feasible to set up a designated NTD unit in all countries, we encourage the articulation of distinct and well-defined NTD-focused programs of work, with activity milestones and goals agreed upon across all systems levels (local, district, national, executive) and increased efforts to ‘mainstream’ [1] NTD activities into any national health infrastructure. Doing this will help rally commitment for NTD programs’ continuity during future disruptive events.

As highlighted by this research, the erosion of long-established community relationships which are critical to NTD programs’ success during the pandemic—particularly through the influence of COVID-19 misinformation–is highly concerning. Previous studies identified the importance of pro-active community education and engagement activities to address both pandemic-related misunderstandings and the associated worker mistrust that can inhibit social mobilisation during routine NTD control activities [19,21]. However, in this study, lengthy emergency public health strategies interrupted established routines and prohibited the traveling and mixing of NTD workers and populations. This largely limited early opportunities to address challenges to health worker-community relationships. In these situations, additional efforts are likely to be needed to rebuild meaningful community engagement before NTD programs can effectively resume. For example, one useful strategy could be creating new forums for exploring emerging concerns regarding interactions between usual NTD medication regimes and new treatments/vaccines being developed and deployed in emergency situations. The COVID-19 pandemic also drew attention to how entrenched social, cultural, and economic inequalities undermined social trust and solidarity, which then facilitated the spread of misinformation and associated mistrust in public services [9,36]. Using community input to redesign returning control programs allows communities to regain a level of efficacy and control over their circumstances, and may help restore socially considerate motivations. As indicated by one example in our study, this would involve giving NTD programs greater flexibility to respond to diverse local preferences and definitions of health needs even when this does not correspond within usual practice scope.

Finally, our study found that systemic weaknesses constrained NTD program capacity to both mount modified responses during the height of the public health restrictions and to promptly re-establish program delivery once able to do so. The range of factors that underpinned these operations across settings included health leadership, budgetary, and administration efficiency, alongside the maintenance of healthcare services with an adequate and trained health workforce. This aligns with the emerging evidence that the underlying strength of health systems supports the resumption of NTD control activity [20] as well as improving overall performance during a global crisis [9,16]. As countries look to improve post-pandemic resilience, our findings support calls for increased attention to whole of health systems strengthening [3,21,24].

Addressing any declining investment in NTD programs as raised by our participants will be an essential first step. In the short term, it will be important to focus on more efficient use of remaining funding to work towards greater activity harmonisation that can reduce duplication and siloed approaches. It will also be critical to explore opportunities to better incorporate NTD management activities within the scope of major global health funders to leverage their human resources, service delivery, and monitoring systems [2, 35] to maintain the crucial work of NTD programs, whatever future emergencies arise.

## Limitations

This study was undertaken in a complex and volatile period, with COVID-19 waves disrupting health service provision and day-to-day life. As such, participants’ recall of the sequence and timing of events may be variable, rendering accurate interpretation challenging for a research team largely unconnected to the daily experience of on-the-ground NTD program operations. The structure of NTD units or programs in the countries included and the diseases for which personnel have responsibility constrained participants’ ability to comment on the full range of NTDs of concern in that setting. This was particularly noticeable in relation to diseases such as dengue, which were often subsumed under a separate vector-borne disease unit. Invitation to participate was circulated through the ACE-NTD network which assisted in identifying potential participants: 11 persons agreed to participate, and seven persons did not respond to follow-up efforts. It is possible that countries that do not have NTD activities connected to this network may not have been informed about the study. Data was collected from individuals operating at national levels and hence may not reflect the full spectrum of frontline service delivery constraints or adaptations performed. Given the small sample size, the conclusions of the study may not be generalisable to other South-East Asian and Pacific countries. Some interviews were conducted in the interviewees’ native language and in translation nuance may have been lost. These limitations notwithstanding, this study adds valuable understanding of adaptive strategies employed across a broader range of countries than has previously been explored. A strength is the study’s duration, which provided the opportunity to understand how NTD control activities evolved over the course of the pandemic.

## Conclusion

Bolstering the capacity of NTD units and staff to respond to the evolving range of challenges presented during widespread disruptive emergencies requires attention to programmatic and structural features. Experiences of participants in this study underscore the role of proactive contingency planning to build on existing strengths, including strong cross-program relationships and adaptability to local contexts, which drove innovative solutions. Results also highlight the importance of supporting these efforts through better recognition and the valuing of NTD control interventions as core health activities, as well as the crucial task of improving the underlying strength of national health systems in which NTD programs operate.

## Supporting information

S1 File Interview guides. (DOCX)

S1 Table Reported NTDs of concern in participating South-East Asian and Pacific countries. (DOCX)
