# Supplementary material for: Understanding how neglected tropical diseases programs in five Asia-Pacific countries adjusted to the COVID-19 pandemic: A qualitative study
Source: PLoS Negl Trop Dis. 2024 May 30;18(5):e0012221. doi: 10.1371/journal.pntd.0012221 (PMC11166303; doi:10.1371/journal.pntd.0012221)
Supplement: S1 File — (DOCX) [file pntd.0012221.s001.docx]

**The EFFECT of COVID-19 on NTD programs: INITIAL ITERVIEW GUIDE**

*Researchers from the NTD Research Group at the Kirby Institute, UNSW Sydney, Australia, are carrying out a study to understand how NTD control activities in countries in the Asia-Pacific region have been affected by the pandemic, and what changes and adaptations were needed to ensure that people affected by NTDs could still access the diagnosis, treatment, and follow-up care they need, despite pandemic restrictions.*

*The reason we are doing this study is to document and share how people have managed to keep NTD control activities going during the pandemic, so we can all learn from each other.*

*There are no right or wrong answers to any of the questions. If there are any questions you aren’t sure about or would prefer not to answer that is absolutely fine.*

*Anything you say will only be used for research purposes and will not be reported back to anyone you work with.*

Blue text involves questions updated when data collection resumed in 2022

**SECTION 1: NTDS IN YOUR COUNTRY**

***Lead-in to this section:*** *This interview is made up of 5 parts. In this first part I’m going to ask you some questions about the NTD control activities in your country.*

1. **Could you please tell me about your job role and your job responsibilities in your organization or team?**

*Explore: Length of time doing this*

*Specifics of role (people management/policy/strategy)*

*Who does role report to*

1. **Within the Ministry of Health in your country, is there a formal NTD unit or department responsible for NTD control activities?**

*Explore: If yes, what is the structure and how many people work in the unit/department?*

*If no, how are NTD control activities implemented, and under which government department?*

*Do you feel this structure is effective?*

*Do you think anything could be improved?*

*Are resources (HR/funding etc) adequate?*

1. **My understanding is that the main NTDs affecting your country are ________. What control activities are in place for these NTDs, and which do you and your team focus on?**

*Explore: How much of a public health problem are these NTDs?*

*How long have they been a problem in your country?*

*Are you aiming to eliminate these NTDs or just control them?*

*How often are these control activities carried out?*

*Are they country-wide activities or localized to specific regions? Which ones?*

*How long have these activities been in place/carried out?*

1. **Do your NTD control activities just cover NTDs, or are there any other infectious diseases also included? Which ones?**

*Explore: How do the activities overlap?*

*Are there different teams responsible for the non-NTD infectious diseases or do you cover these?*

**SECTION 2: IMPACT OF COVID-19 PANDEMIC ON NTD CONTROL ACTIVITIES**

***Lead-in to this section:*** *In this second section of the interview, I’m going to ask you some questions about how the pandemic has affected your country and specifically the NTD control activities you have described*

1. **Can you tell me about the COVID-19 pandemic in your country? What public health strategies were put in place throughout the pandemic ?**

*Explore: Types of restrictions and duration of restrictions
When restrictions started/ended/repeated
Case numbers and deaths
When cases/deaths started
Vaccination status in country*

*Impact of multiple strains/waves (if relevant)*

1. **In your opinion, have these measures been effective? Are communities taking them seriously, and what are the consequences for not complying with measures?**

*Explore: People’s understanding of COVID*

*Compliance with restrictions*

*Response to relaxing of restrictions and re-opening of travel/movement pathways (if relevant)*

*Do you think these measures go far enough to control the pandemic in your country?*

1. **Thinking about each NTD control activity you are involved with, in your opinion, have these been negatively impacted by the pandemic and how? For example, changes in how/when MDA is delivered/funding/drug availability/staffing issues?**

*Explore: Impact on MDA/WaSH/vector control/morbidity management*

*Shipment of drugs; drug delivery to people/communities
 Lockdown restrictions
 Long-term care programs/support
 Program funding
 Staffing issues (trained/untrained medical staff and community volunteers) e.g., redeployment*

**SECTION 3: ADAPTATION, INNOVATION, and INTEGRATION**

***Lead-in to this section:*** *In this third section of the interview I am* *going to ask you some questions about how you and your team have tried to overcome any issues you’ve had with delivering NTD control activities during the pandemic, and if you have needed to make any changes to how you deliver these programs.*

1. **Thinking about each NTD control activity you are involved with, how did you overcome any issues you had delivering these due to the pandemic?**

*Explore: Successes, challenges, and failures
Were there activities you couldn’t deliver as planned and why? ~~Do you think you will be able to deliver these soon?~~*

*Which aspects of the program were modified and how?*

*Did approaches change if there were multiple waves (such as a surge in new cases linked to a new variant)?*

1. **Do you believe any of the modifications that you made to your NTD control activities as a result of the pandemic were particularly innovative?**

*Explore: Drug delivery methods to individuals/the community
Integration with other NTD programs/funding/staffing etc
Delivery of long-term care in the community*

*Use of risk management frameworks to inform decision making/redeployment
How did you come up with these ideas for modifications?
If no, did you think about doing anything innovative?*

1. **Did you partner with any other organizations or other programs that you wouldn’t normally work with in order to deliver your NTD control activities during the pandemic? How did this partnership work?***Explore: Which types of organization? E.g. Faith-based organizations, community-based organizations*

*What activities did you collaborate on and how?*

*How did you come up with this idea to collaborate?
 What went well and what didn’t go so well with the collaboration?*
 *Was this collaboration beneficial? Would you do it again or do anything differently?*

1. **Have you received any feedback – either positive or negative – from the community on how your NTD control activities were delivered during the pandemic?**

*Explore: If not, did you engage with the community in delivering NTD activities during the pandemic?*

*If there were changes to activities because of the pandemic, did you involve the community?*

*Do you plan to collect feedback from communities about NTD control activities?*

1. **Do you agree with the interruptions or restart of NTD control activities in your country? If activities have restarted do you feel whether you have been supported to safely managed these, given the ongoing pandemic?**

*Explore: If not, what do you think should have been done differently?*

*What aspects do you agree with, and what don’t you agree with?*

*What do you think should have been done differently?*

*Do you think this is view that others in your department/team share or just you?*

**SECTION 4: WATER, SANITATION, AND HYGIENE IMPACT DURING THE PANDEMIC**

***Lead-in to this section:*** *In this next section of the interview, I am going to ask you some questions about about water, sanitation, and hygiene (WaSH) activities in your country. Given hand hygiene is the main strategy for preventing transmission of COVID-19, and that improving WaSH access and infrastructure a control strategy for some NTDs, we are interested to know how the pandemic has impacted on WaSH activities in your country.*

**What role does WaSH access/health promotion in the control of any NTDs that are prevalent in your country?***Explore: Which NTDs?*

*Infrastructure or health promotion or both?*

*How important do you think WASH is vs other control activities for these NTDs?*

1. **Are there any WaSH activities in place in your country?**

*Explore: Do these exist mainly to support NTD control activities, or infrastructure improvements, or both?*

*Do you think these activities are adequate? If not, what could be improved/changed?*

1. **Are you and your team involved in managing any of these WaSH activities or are they managed by another team?**

*Explore: Which other team?*

*Does this arrangement work?*

*Are there any challenges?*

1. **Have any aspects of these WaSH activities changed because of the pandemic?**

*Explore: Funding*

*Awareness of the importance of handwashing*

*Key messages or emphasis in health promotion campaigns*

*Have you noticed any changes in attitude to WASH in communities resulting from the pandemic?*

**SECTION 5: SUSTAINABILITY OF PROGRAM CHANGES AFTER THE PANDEMIC**

***Lead-in to this section:*** *In this final section of the interview I’m going to ask you some questions about whether you think any of these changes will carry on after the pandemic ends, especially if the changes have been successful.*

1. **To what extent do you think any changes/adaptations to your NTD control activities will continue after the pandemic ends/ To what extent do you think any changes/adaptations to your NTD control activities will continue as the COVID response globally appears to be moving from elimination to disease control?**

*Explore: Changes to keep; changes to discard
Challenges with keeping these changes
Challenges and benefits of continuing with adaptations to program*

1. **What do you think are your most important learning points – both positive and negative – from operating your NTD control activities during a pandemic? Would you have done anything differently?**

*Explore: Learning points, successes, challenges
Anything you would have done differently?*

1. **Is there anything else you would like to add?**

**The EFFECT of COVID-19 on NTD programs: FOLLOW-UP INTERVIEW GUIDE**

*12 months ago, you participated in an interview with researchers from the NTD Research Group at the Kirby Institute, UNSW Sydney, Australia, in a study to understand how NTD control activities in countries in the Asia-Pacific region have been affected by the pandemic, and what changes and adaptations (if any) were needed to ensure that people affected by NTDs could still access the diagnosis, treatment, and follow-up care they need, despite pandemic restrictions.*

*The research team is interested in finding out about the impact of pandemic since we last spoke, including the effect of the vaccine rollout, as well as any new changes you have needed to make to respond to new developments in the pandemic.*

*There are no right or wrong answers to any of the questions. If there are any questions you aren’t sure about or would prefer not to answer that is absolutely fine.*

*Anything you say will only be used for research purposes and will not be reported back to anyone you work with.*

**SECTION: IMPACT OF COVID-19 PANDEMIC ON NTD CONTROL ACTIVITIES**

***Lead-in to this section:*** *In this section of the interview, I’m going to ask you some questions about how the pandemic has affected your country and specifically the NTD control activities you have described*

1. **Since we last spoke in [month/year] can you tell me about any further impacts from the COVID-19 pandemic in your country? Were there any new public health strategies that were put in place throughout the pandemic ?**

*Explore: Types of restrictions and duration of restrictions
When restrictions started/ended/repeated
Case numbers and deaths
When cases/deaths started
Vaccination status in country*

*Impact of multiple strains/waves (if relevant)*

1. **In your opinion, have these new measures been effective? Are communities taking them seriously, and what are the consequences for not complying with measures?**

*Explore: People’s understanding of COVID*

*Compliance with restrictions*

*Response to relaxing of restrictions and re-opening of travel/movement pathways (if relevant)*

*Do you think these measures go far enough to control the pandemic in your country?*

1. **Thinking about each NTD control activity you are involved with, in your opinion, have these been further negatively impacted by the pandemic since we last spoke and how? For example, changes in how/when MDA is delivered/funding/drug availability/staffing issues?**

*Explore: Impact on MDA/WaSH/vector control/morbidity management*

*Shipment of drugs; drug delivery to people/communities
 Lockdown restrictions
 Long-term care programs/support
 Program funding
 Staffing issues (trained/untrained medical staff and community volunteers) e.g., redeployment*

**SECTION: ADAPTATION, INNOVATION, and INTEGRATION**

***Lead-in to this section:*** *In this next section of the interview I am* *going to ask you some questions about how you and your team have tried to overcome any issues you’ve had with delivering NTD control activities during the pandemic, and if you have needed to make any changes to how you deliver these programs.*

1. **Thinking about each NTD control activity you are involved with, how did you overcome any issues you had delivering these due to the pandemic since we last spoke? This could be a new strategy or adaptation you have tried, or using a previous successful strategy to respond to a new situation, like a new COVID variant or the vaccine rollout.**

*Explore: Successes, challenges, and failures
Where there activities you couldn’t deliver as planned and why?*

*Which aspects of the program were modified and how?*

*Did approaches change if there were multiple waves (such as a surge in new cases linked to a new variant)?*

1. **Do you believe any of the new modifications that you made to your NTD control activities as a result of the pandemic were particularly innovative?**

*Explore: Drug delivery methods to individuals/the community
Integration with other NTD programs/funding/staffing etc
Delivery of long-term care in the community*

*Use of risk management frameworks to inform decision making/redeployment, such as those suggested by the WHO?
How did you come up with these ideas for modifications?
If no, did you think about doing anything innovative?*

1. **Since we last spoke, have you partnered with any other organizations or other programs that you wouldn’t normally work with in order to deliver your NTD control activities during the pandemic? How did this partnership work?***Explore: Which types of organization? E.g. Faith-based organizations, community-based organizations*

*What activities did you collaborate on and how?*

*How did you come up with this idea to collaborate?
 What went well and what didn’t go so well with the collaboration?*
 *Was this collaboration beneficial? Would you do it again or do anything differently?*

1. **Since we last spoke, have you received any feedback – either positive or negative – from the community on how your NTD control activities were delivered during the pandemic?**

*Explore: If not, did you engage with the community in delivering NTD activities during the pandemic?*

*If there were changes to activities because of the pandemic, did you involve the community?*

*Do you plan to collect feedback from communities about NTD control activities?*

**SECTION: WATER, SANITATION, AND HYGIENE IMPACT DURING THE PANDEMIC**

***Lead-in to this section:*** *In this next section of the interview, I am going to ask you some questions about about water, sanitation, and hygiene (WaSH) activities in your country. Given hand hygiene is the main strategy for preventing transmission of COVID-19, and that improving WaSH access and infrastructure a control strategy for some NTDs, we are interested to know how the pandemic has impacted on WaSH activities in your country.*

1. **Since we last spoke, are you aware of any aspects of WaSH activities that have changed because of the pandemic?**

*Explore: Funding*

*Awareness of the importance of handwashing*

*Key messages or emphasis in health promotion campaigns*

*Have you noticed any changes in attitude to WASH in communities resulting from the pandemic?*

**SECTION: SUSTAINABILITY OF PROGRAM CHANGES AFTER THE PANDEMIC**

***Lead-in to this section:*** *In this final section of the interview I’m going to ask you some questions about whether you think any of these changes will carry on after the pandemic ends, especially if the changes have been successful.*

1. **To what extent do you think any changes/adaptations to your NTD control activities will continue as the COVID response globally appears to be moving from elimination to disease control?**

*Explore: Changes to keep; changes to discard
Challenges with keeping these changes
Challenges and benefits of continuing with adaptations to program*

1. **Thinking particularly about what you have done in the last [number of] months since we spoke, what do you think are your most important learning points – both positive and negative – from operating your NTD control activities during a pandemic? Would you have done anything differently?**

*Explore: Learning points, successes, challenges
Anything you would have done differently?*

1. **Is there anything else you would like to add?**
