# Supplementary material for: Understanding how neglected tropical diseases programs in five Asia-Pacific countries adjusted to the COVID-19 pandemic: A qualitative study
Source: PLoS Negl Trop Dis. 2024 May 30;18(5):e0012221. doi: 10.1371/journal.pntd.0012221 (PMC11166303; doi:10.1371/journal.pntd.0012221)
Supplement: S1 Table — (DOCX) [file pntd.0012221.s002.docx]

**Supplementary Table 1. Reported NTDs of concern in participating South-East Asian and Pacific countries^a^**

|  | Fiji | Papua New Guinea | Philippines | Timor-Leste^c^ | Vanuatu |
| --- | --- | --- | --- | --- | --- |
| Buruli ulcer |  | Endemic |  |  |  |
| Dengue | Endemic | Endemic | Endemic | Endemic | Endemic |
| Food-borne Trematodes |  |  | Endemic |  |  |
| Leprosy | Eliminated | Eliminated | Eliminated | Endemic | Eliminated |
| Lymphatic Filariasis (LF) | Endemic | Endemic | Endemic | Endemic | Eliminated |
| Rabies |  |  | Endemic |  |  |
| Scabies | Endemic | Endemic |  | Endemic | Endemic |
| Schistosomiasis |  |  | Endemic |  |  |
| STH | Endemic | Endemic | Endemic | Endemic | Endemic |
| Trachoma^b^ | Present, PH  importance  unclear | Endemic |  |  | Eliminated |
| Yaws |  | Endemic | Endemic | Endemic | Endemic |

^a^ As reported by participants with additional information adapted from [1-8]. Eliminated: Eliminated as a public health problem; Endemic: interventions needed or ongoing.

^b^ Defined according to prevalence of trachomatous inflammation – follicular (TF).

^c^ Timor-Leste is planning surveillance to confirm elimination of LF and interruption of yaws transmission [2, 4].

**References**

1. World Health Organization Regional Office for the Western Pacific. Regional action framework for control and elimination of neglected tropical diseases in the Western Pacific [Internet]. Manila, Philippines: World Health Organization Regional Office for the Western Pacific; 2020 Apr 28 [cited 2023 Jan 30]. Available from: <https://www.who.int/publications/i/item/9789290619079>.

2. da Costa Martins NE, Jung Y, Joao JC, da Silva ES, Kim SH, dos Santos MA, et al. Transmission Assessment Survey in Timor-Leste Lymphatic Filariasis, Soil-Transmitted Helminthiasis, Yaws, and Scabies: 2020-2021. Dili: Ministerio de Saude, World Health Organization Timor Leste, Korea International Cooperation Agency; June 2022. 124p.

3. Handley BL, Roberts CH, Butcher R. A systematic review of historical and contemporary evidence of trachoma endemicity in the Pacific Islands. PLoS One. 2018;13(11):e0207393.

4. Yajima A, Lin Z, Mohamed AJ, Dash AP, Rijal S. Finishing the task of eliminating neglected tropical diseases (NTDs) in WHO South-East Asia Region: promises kept, challenges, and the way forward. The Lancet Regional Health - Southeast Asia. 2023;18.

5. Kazadi WM, Asiedu KB, Agana N, Mitjà O. Epidemiology of yaws: an update. Clin Epidemiol. 2014;6:119-28.

6. Togami E, Chiew M, Lowbridge C, Biaukula V, Bell L, Yajima A, et al. Epidemiology of dengue reported in the World Health Organization's Western Pacific Region, 2013-2019. Western Pac Surveill Response J. 2023;14(1):1-16.

7. Matthews RJ, Kaluthotage I, Russell TL, Knox TB, Horwood PF, Craig AT. Arboviral disease outbreaks in the Pacific Islands countries and areas, 2014 to 2020: a systematic literature and document review. Pathogens. 2022;11(1).

8. Leonardo L, Hernandez L, Magturo TC, Palasi W, Rubite JM, de Cadiz A, et al. Current status of neglected tropical diseases (NTDs) in the Philippines. Acta Trop. 2020 Mar;203:105284.
